# Editorial: Cytokine signaling and innate host defense in modulation of viral infections and the viral evasion

**DOI:** 10.3389/fcimb.2025.1694793

**Published:** 2025-09-15

**Authors:** M. Victoria Delpino, Jorge Quarleri

**Affiliations:** Universidad de Buenos Aires (UBA), Consejo de Investigaciones Científicas y Técnicas (CONICET), Instituto de Investigaciones Biomédicas en Retrovirus y Sida (INBIRS), Laboratorio de Inmunopatogénesis Viral, Buenos Aires, Argentina

**Keywords:** cytokine signaling, innate immunity, viral evasion, immunometabolism, host-pathogen interaction

Viral infections do more than passively replicate within host cells. They reprogram cellular metabolism, alter immune signaling pathways, and reshape tissue homeostasis. These changes influence both the acute manifestations of disease and long-term health outcomes (Heaton and Randall, 2011; Sanchez and Lagunoff, 2015; Thaker et al., 2019). The articles in this Research Topic explore how viruses and the body’s defenses interact, how viruses get around the body’s defenses that are triggered by cytokines produced after sensing the infection, and how we might use this information to come up with better ways to diagnose and treat infections.

A common thread is how infections change with the cell’s metabolism and what happens to the cell due to the changes. Cevallos et al. show that SARS-CoV-2 infection of hepatocytes disrupts mitochondrial homeostasis and lipid metabolism, promoting ferroptosis as a big factor in how the virus causes disease. It also suggests that controlling mitochondrial ROS and iron might help reduce liver problems tied to infection. Gao et al. add to this by reporting that chronic patients on antiviral therapy have changed metabolism Their work connects changes in metabolites with changes in cytokine networks, which suggests that immunometabolism can serve as a biomarker and regulator of antiviral immunity.

The importance of metabolism in viral persistence is supported by Freiberger et al., who show that HIV infection can push mesenchymal stromal cells toward becoming proinflammatory adipocyte-like. This state involves changes in the way of how the body handles lipids and releases inflammatory mediators, which sheds light on why people living with the problem experience metabolic and adipose tissue issues.

Taken together, these papers emphasize the strong interconnection between metabolic pathways and innate immunity and showed that metabolic pathways decide how cytokine networks are turned on, reshaped, or thrown off balance during short-term and long-term infections.

Innate immunity doesn’t act the same way everywhere in the body, and how organs respond to cytokines is another topic covered here. Wu et al. highlights GPR4 antagonism as a promising therapeutic avenue in severe COVID-19. Using a SARS-CoV-2-infected K18-hACE2 mouse model, the authors show that pharmacological inhibition of GPR4 improves survival, attenuates cytokine-driven inflammation, and reduces viral burden in both lung and brain tissues. These findings position GPR4 blockade as a potential dual-action strategy—modulating detrimental inflammation while limiting viral replication—that warrants further investigation for COVID-19 and related viral diseases.

These results show that tweaking the body’s inflammatory sensors could lead to treatments that help with acute infection. At the same time, Gandhi et al. underscore the value of biomarker-based screening to detect subclinical renal injury in patients with chronic hepatitis B (CHB). By analyzing serum and urinary proteins in treatment-naïve individuals, the authors identify several informative indicators, with a combined biomarker panel achieving high sensitivity and specificity for early kidney injury. Importantly, this approach allows detection of renal impairment before conventional markers such as serum creatinine become abnormal, offering a noninvasive tool to improve risk stratification and prognosis in CHB. This highlights how immune activation and interactions between the virus and the body can quietly hurt organs before symptoms show up.

These articles together stress that cytokines are a double-edged sword: they’re needed to keep viruses in check, but they can be harmful if they’re overactivated or misdirected.

At the molecular level, some articles in this section highlight the interaction back-and-forth between the body’s virus sensors and how viruses get around them is also discussed. Yuan et al. review the emerging importance of ubiquitin-like (UBL) modifications in shaping HIV–host interactions. Beyond acting as simple antiviral defenses or viral tools, UBLs play dual roles by regulating both host restriction factors and viral proteins, thereby influencing HIV replication, immune evasion, and intracellular signaling. By synthesizing recent advances, the authors position UBLs as central players in the ongoing virus–host arms race and as potential targets for innovative therapeutic strategies against HIV by pointing host pathways that are less likely to be escaped by the virus as a treatment approach.

Other papers back up how viruses interfere with innate immune pathways. Wang et al. uncovers a novel mechanism by which infectious bursal disease virus (IBDV) undermines host antiviral defenses in chickens. The authors demonstrate that the viral VP3 protein interacts with and promotes proteasome-mediated degradation of IRF7, thereby suppressing type I interferon signaling and facilitating viral replication. Notably, this effect is pronounced with very virulent IBDV strains but absent in attenuated forms. These findings highlight IRF7 as a pivotal target of viral immune evasion and provide new insights into IBDV pathogenesis, with potential implications for vaccine design and antiviral strategies in poultry health.

Similarly, Liu et al. show an epitope-based nanoparticles (NPs) as a promising next-generation vaccine strategy against SARS-CoV-2. By incorporating conserved spike protein epitopes into ferritin-based NPs, the authors achieved durable antibody responses, neutralizing activity, balanced Th1/Th2 cellular immunity, and antibody-dependent cellular cytotoxicity in animal models. These findings highlight the potential of epitope-focused nanovaccines to overcome challenges posed by spike protein variability, offering a path toward broader and more durable protection against COVID-19.

The interaction between innate immunity and other comorbidity is shown at the clinical study level. Alalwan et al. identifies type III interferons as key modulators of COVID-19 severity. While IFNλ4 expression showed no association with disease outcome, elevated IFNλ2 levels correlated strongly with severe disease, independent of major demographic and clinical factors. Notably, this association was evident only in non-obese individuals, suggesting that obesity may obscure IFNλ2’s predictive value. These findings highlight IFNλ2 as a potential biomarker of COVID-19 severity and underscore the need to consider host metabolic status when evaluating immune correlates of disease progression.The study clearly highlights the importance of the translational studies, and reminds that body physiology—whether metabolic, inflammatory, or structural— changes the outcome of infections as much as viral make up does.

When put together, this section shows a connected view of viral immunopathogenesis. Pathogens alter metabolic pathways, hijack post-translational changes and modify stromal or adipose cell identities. These aren’t separate problems. They spread through immune networks and organ function, creating both risks for pathology and chances for intervention. Some works in this Research Topic show how metabolic or cytokine biomarkers can be used for diagnosis, while others pinpoint therapeutic spots where changing the body’s response could lessen viral disease.

A unifying idea from this section is that cytokine signaling and innate host defense are inherently double-edged. They offer the necessary means by which pathogens are recognized and controlled. At the same time, their dysregulation contributes to tissue injury, systemic inflammation, and long-term issues. Viral evasion strategies—from degradation of IRF7 to subversion of UBL signaling—remind us that pathogens get around this balance for their own gain, keeping up persistence or increasing spread.

All these studies bring up key questions. Can metabolic pathways such as tryptophan catabolism or lipid remodeling be tweaked to adjust cytokine responses without causing pathology? How might organ-specific changes of innate sensors, such as GPR4 inhibition, be used to reduce damage in acute infections? What vaccine strategies will do the best job of predicting viral change while knowing about immune evasion? Maybe most importantly, how do the health issues—such as obesity to chronic viral carriage—reshape innate immunity in ways that change how likely we are to catch new viral threats?

The works in this Research Topic give not only mechanical depth but also translational hope. By showing how viruses interact with cytokine signaling and innate defense, they map out paths toward accurate immunomodulation—approaches that try to boost protective immunity while lowering damage to tissues.
